# An update of fire needle acupuncture for acute herpes zoster and prevention of postherpetic neuralgia in adults: A protocol for systematic review and meta-analysis

**DOI:** 10.1097/MD.0000000000024180

**Published:** 2021-01-08

**Authors:** Jiaxuan Wang, Xindong Wang, Hongyan Xia, Na Zhang, Shiyu Lin, Jingchun Zeng, Guohua Lin

**Affiliations:** a Guangzhou University of Chinese Medicine; b The First Affiliated Hospital of Guangzhou University of Chinese Medicine, Guangzhou, 510405, China.

**Keywords:** acupuncture, burn needle, fire needle, herpes zoster, PHN, postherpetic neuralgia

## Abstract

**Background:**

Herpes zoster, is one of the most familiar skin diseases in conducted a systematic review and meta-analysis in 2018 showed that fire needle acupuncture can relieve the pain caused by herpes zoster quickly and prevent the outcome of postherpetic neuralgia (PHN), with little side effects. The purpose of this study is to update the systematic review with the latest evidence.

**Methods:**

Four English (PubMed, Embase, the Cochrane Library, the Web of Science) and 4 Chinese databases (CNKI, Wanfang, VIP, and CBM) will be searched dating until 30 June 2020 for randomized controlled trials with no language restrictions. In addition, a hand search of the reference lists of included studies will also be done. Adults (aged 18–70) with acute herpes zoster (less than 7 days) using fire needle acupuncture will be included. Pairs of researchers will independently conduct the search, screen titles and abstracts, retrieve full texts of potentially eligible studies, assess the risk of bias, and conduct date extraction and synthesis. If there is any discrepancy in the whole process, consult a third researcher. For meta-analysis, the primary outcome is the pain intensity (visual analogue scale [VAS] pain scale; pain relieve of 30%, duration of pain), and the second outcome is incidence of PHN. A sequential analysis will be done to test the robustness of results of meta-analysis. The quality of evidence will be assessed using GRADE system.

**Results:**

The results will be published in a peer-reviewed journal.

**Conclusion:**

This study will provide the latest systematic review and meta-analysis of fire needle acupuncture for acute herpes zoster and prevention of PHN.

## 1 Introduction

Herpes zoster, also called shingles, is one of the most familiar skin diseases in adults. A systematic review published in 2014 reported an incidence of 3 to 5/1000 person years for herpes zoster, with an increased incidence of 6 to 8/1000 person year at 60s and 8 to 12/1000 person year at 80s.^[1]^ The main manifestations were cluster herpes and severe neuralgia along the nerve distribution area. It is currently recognized that the varicella zoster viruses stay latent in the ganglia after infection, and become active again when the body's immunity is weakened. The main treatment methods include antiviral drugs, nourishing nerves drugs, anti-inflammatory drugs, and pain relief drugs. However, after the full course of above treatment, many patients still feel pain, lasting more than 1 month or longer.^[2,3]^ This may be because the nerve damage caused by the virus is difficult to recover and manifests as postherpetic neuralgia (PHN), which is the most severe and common sequelae of herpes zoster, characterized by severe, constant, intermittent burning, or lancinating pain with allodynia.^[4,5]^ At present, PHN has been recognized as a kind of neuropathic pain, and hard to cure. Therefore, early, sufficient, full course, and combined treatment have become the important principle for the treatment of herpes zoster.^[6]^

Herpes zoster belongs to the category of “lumbar fire stripe” or “snakelike herpes” in traditional Chinese medicine (TCM). According to the TCM theory of “Removing the Stagnation of Fire,” we can use fire needle acupuncture to treat herpes zoster in China.^[7,8]^ At present, some small sample randomized controlled trials have confirmed that fire needle acupuncture can shorten the duration of pain, reduce the incidence of PHN, and has few adverse effects.^[9,10]^ However, a single study has problems such as small sample size and low quality of literature, limiting its reference significance. We conducted a systematic review and meta-analysis of fire needle acupuncture on acute herpes zoster in 2018 (search dated until Nov 2018).^[11]^ Four randomized controlled trials with different outcomes have published since the last search.^[12–15]^ Almost 2 years have passed, so we decide to update the last systematic review. This review aims to systematic review existing evidences for fire needle acupuncture treating acute herpes zoster and prevention of postherpetic neuralgia. We will conduct this protocol with reference to participants, interventions, comparators, and outcomes (PICO) principles.

## 2 Methods and analysis

The study protocol has been developed based on the Preferred Reporting Items for Systematic Reviews and Meta-Analyses for Protocols (PRISMA-P) guidelines.^[16]^

### 2.1 Search strategy

#### 2.1.1 Databases and timeframes

Four English (PubMed, Embase, the Cochrane Library, the Web of Science) and 4 Chinese databases (China national knowledge infrastructure (CNKI), Wanfang Data E-Resources (Wanfang), VIP Citation Database (VIP), and China Biology Medicine (CBM)) will be searched dating until November 2020 with no language restrictions, and translations will be sought where necessary.

#### 2.1.2 Search strategy

We (JW and XW) will conduct the search in the databases mentioned above. Any discrepancies will be solved via discussion or, if necessary, by consulting a third researcher (GL). The search aims to find both published and unpublished publications. A two-step strategy will be used.

1. First search: All databases mentioned above will be searched in English and Chinese. The search terms including herpes zoster, fire needle, PHN, postherpetic neuralgia, RCT, etc will be involved in the search strategy (Keywords or Title/Abstract or MeSH terms). A detailed search strategy in PubMed is described as an example as follows “((acupuncture) OR (fire needle) OR (red hot needle) OR (burn needle)) AND ((herpes zoster) OR (shingles) OR (postherpetic neuralgia) OR (PHN) OR (herpetic neuralgia)) AND ((RCT) OR (randomized controlled trial) OR (trial)).”
2. Second search: The reference list of included articles will be manually examined to identify additional studies for inclusion in the systematic review.

Finally, we will rerun the search just before data synthesis to identify any new studies that should be retrieved for inclusion.

### 2.2 Types of studies to be included

Studies will be included so long as inclusion criteria are met. The eligibility criteria are summarized using the PICOS approach (patients/participants, intervention, comparisons/control, outcomes, and study design type).

#### 2.2.1 Inclusion criteria

1. Types of studies: Randomized controlled trials (RCTs).
2. Patients: Adults with acute herpes zoster (less than 7 days) from 18 to 70 years old. There are no restrictions on ethnic distribution and gender.
3. Interventions: Fire needle acupuncture alone or mild assistance with other acupuncture techniques such as electroacupuncture, cupping, moxibustion, bloodletting, etc.
4. Control: Conventional chemical drugs for acute herpes zoster.
5. Outcome: Pain intensity or incidence of PHN.

#### 2.2.2 Exclusion criteria

1. Editorials, letters, protocols, case reports, controlled clinical trials, and conference proceedings.
2. Repeated articles or translated articles.
3. Full-text article not available, abstracts or literature with incomplete data that can be extracted.
4. Fire needle acupuncture combined with chemical drugs or traditional Chinese medicine.

### 2.3 Condition or domain being studied

Pain intensity after treatment and incidence of PHN.

### 2.4 Participants/population

Inclusion: Adults (18–70 years old) diagnosed with acute herpes zoster (less than 7 days) using criteria such as the International Classification of Diseases and Related Health Problems (ICD-10) or other certified diagnostic criteria. There are no restrictions on ethnic distribution and gender.

Exclusion: Special groups such as pregnant and lactating women; patients with severe cardiovascular or cerebrovascular diseases; patients with non-shingles-induced peripheral nerve damage with paranesthesia; patients with severe mental illness, etc.

### 2.5 Interventions

Fire needle acupuncture (a method of quickly piercing the skin of human with needles which have burned red hot on an alcohol lamp to treat multiple diseases) alone or mild assistance with other acupuncture techniques such as electroacupuncture, cupping, moxibustion, bloodletting, etc. Acupoint selection, operation time, and manipulation are not limited.

### 2.6 Control

Conventional chemical drugs for acute herpes zoster, including but not limited to antiviral drugs (acyclovir, valaciclovir, etc), neurotrophic drugs (VitB6, VitB12, etc), non-steroidal anti-inflammatory drugs (celebrex, loxonin, etc), etc. The dosage form and treatment course are not limited.

### 2.7 Outcomes

The primary outcome is pain intensity, including visual analogue scale (VAS) pain scale, pain relief time of 30%, and duration of pain because these are the most concern of patients.

The second outcome is incidence of PHN. PHN is broadly defined as clinically relevant pain or altered sensation persisting in the regions affected by herpes zoster at least 3 months beyond the initial herpes zoster eruption.^[17]^ We will also focus on the side effects during the review.

### 2.8 Study screening

Yields from searches will be exported into the reference manager software EndNote X9.1. Duplicates will be removed. Two researchers (JW and XW) will independently screen study titles and abstracts against inclusion and exclusion criteria. Any discrepancies will be solved via discussion or, if necessary, by consulting a third researcher (GL). Excluded study numbers will be dated.

Full-text records of the included studies from the first round of screening are then retrieved. Then, all full-text records retrieved above will be independently assessed against inclusion and exclusion criteria by 2 researchers (JW and XW). Reasons for exclusion will be documented. Any disagreements will be resolved via discussion. A third researcher (GL) will be consulted if consensus is not reached.

Study screening process will be illustrated as a standard PRISMA flow diagram.

### 2.9 Data extraction

Data extraction will be conducted independently by 2 researchers (JW and XW). We will set up a piloting Excel spreadsheet using standardized extraction criteria for all included studies. Each data item will be compared, and any discrepancies will be resolved by discussion, or by adjudication from a third researcher (GL).

For every included study, the following data will be extracted:

- Authors.
- Publication year.
- Title.
- Number of patients, mean age, percentage of men and women (intervention and control group).
- Onset of disease (intervention and control group).
- Therapeutic method (intervention and control group).
- Course of treatment (intervention and control group).
- Outcomes (VAS pain scale, pain relief time of 30%, duration of pain, and incidence of PHN).
- Funding sources.

### 2.10 Risk of bias assessment

Two researchers (HX and NZ) will independently assess the risk of bias of the included studies using the Cochrane Risk of Bias tool (RoB).^[18]^ The RoB tool prompts judgements regarding biases in 5 domains: random sequence generation, allocation concealment, blinding of participants and personnel, blinding of assessment, incomplete outcome data, selective reporting, and other biases. Each domain will be judged by the level of risk of bias: high level, low level, or unclear level. Any disagreements will be solved by discussion or a third researcher's adjudication (GL).

### 2.11 Strategy for data synthesis

A narrative synthesis approach will initially be used to systematically describe the characteristics and quantitative data from the included studies. The continuous variables (VAS pain scale, pain relief time of 30%, duration of pain) will be represented by standard mean differences (SMD), while count data (the incidence of PHN) will be represented using relative risk (RR), and for both, a 95% confidence interval (CI) will be calculated. *P* < .05 will be statistically significant.

### 2.12 Meta-analysis

We will use RevMan 5.3.5 software to conduct meta-analysis of outcomes.^[19]^ Heterogeneity will be assessed using the *χ*^2^ test (*P*-value) and the *I*^2^ statistic. Values of *P* > .1 will be regarded as low heterogeneity, while *P* < .1 will be regarded as high heterogeneity. Then we will use *I*^2^ to estimate the degree of heterogeneity, and will regard *I*^2^ values of 0% to 50% as mild heterogeneity, 50% to 100% as high level of heterogeneity which is not appropriate for making a quantitative meta-analysis. If quantitative synthesis is not appropriate, we will objectively state the results.

Considering the different location of herpes zoster resulting different operating position of every patients, and difficulties to perform blinding to patients and doctors, random effect model will be used for the calculation to reduce the probability of false positive and lower the chance of heterogeneity.

We will try to figure out the source of heterogeneity using sensitivity or subgroup analysis if necessary.

### 2.13 Sensitivity or subgroup analysis

Sensitivity analysis will be done to assess the robustness of the results by excluding studies with high risk of bias or high weighted studies. We aim to carry out subgroup analysis to explore heterogeneity between studies. If possible, the subgroup analysis will be based on:

- Age of patients (18–50 years old, 50–70 years old).
- Onset of disease (1–3 days, 4–7 days).
- Course of treatment (1–10 days, 10 days, and above).
- Therapeutic method (fire needle acupuncture alone, fire needle acupuncture combined with other acupuncture techniques).

### 2.14 Meta-biases

Funnel plots will be used to detect the potential reporting biases if more than 10 studies are included. The Egger's test will be used to determine funnel plot asymmetry.

### 2.15 Sequential analysis

We (JW and XW) will use TSA 0.9.5.10 Beta software to acquire required information size (RIS).^[20]^ Doing this test, we can figure out if we have enough sample sizes for the results of meta-analysis. This will make the results more robust and provide a termination standard for clinical trials, avoiding wasting research funds and medical resources.

### 2.16 Confidence in cumulative evidence

We (SL and JZ) will assess the quality of evidence based on the Grading of Recommendations Assessment, Development, and Evaluation (GRADE) system.^[11]^ The evidence will be adjusted to 4 levels: high, moderate, low, or very low.

### 2.17 Anticipated or actual start date

1 July 2020.

### 2.18 Anticipated completion date

31 Dec 2020.

## 3 Discussion

At present, the treatment of acute herpes zoster is still based on chemical drugs world widely. However, the existing drugs have multiple side effects and none of them but vaccine can reduce the incidence of PHN.^[21]^ Fire needle acupuncture for acute herpes zoster has shown its advantages in several small-scale clinical randomized controlled trials in China, that is, quick pain relief, little side effects, and low incidence of PHN. We made a systematic review and meta-analysis of fire needle acupuncture on acute herpes zoster in 2018,^[11]^ and decide to update it because more trials have been published in 2 years. Therefore, the purpose of this article is to objectively evaluate the effectiveness of fire needle acupuncture for acute herpes zoster and its preventive effect on postherpetic neuralgia. This will provide another choice for doctors and patients.

## Author contributions

All protocol authors read, provided feedback, and approved the final manuscript.

**Conceptualization:** Jiaxuan Wang, Guohua Lin.

**Date extraction and meta-analysis:** Jiaxuan Wang, Xindong Wang.

**GRADE assessment:** Shiyu Lin, Jingchun Zeng.

**Risk of bias assessment:** Hongyan Xia, Na Zhang.

**Searching and screening:** Jiaxuan Wang, Xindong Wang.

**Sequential analysis:** Jiaxuan Wang, Xindong Wang.

**Supervision:** Guohua Lin.

**Writing – draft:** Jiaxuan Wang.

**Writing – review & editing:** Guohua Lin.

## References

[1] Kawai K Gebremeskel BG Acosta CJ Systematic review of incidence and complications of herpes zoster: towards a global perspective. BMJ Open 2014;4:e004833 DOI 10.1136/bmjopen-2014-004833. 10.1136/bmjopen-2014-004833 PMC4067812 24916088

[2] Schmader K Herpes zoster. Clin Geriatr Med 2016;32:539–53. DOI 10.1016/j.cger.2016.02.011. 27394022 10.1016/j.cger.2016.02.011

[3] Koshy E Mengting L Kumar H Epidemiology, treatment and prevention of herpes zoster: a comprehensive review. Indian J Dermatol Venereol Leprol 2018;84:251–62. DOI 10.4103/ijdvl.IJDVL_1021_16. 29516900 10.4103/ijdvl.IJDVL_1021_16

[4] Wehrhahn MC Dwyer DE Herpes zoster: epidemiology, clinical features, treatment and prevention. Aust Prescr 2012;35:143–7.

[5] Wollina U Machetanz J Herpes zoster and postherpetic neuralgia. Hautarzt 2016;67:653–65. DOI 10.1007/s00105-016-3834-y. 27389412 10.1007/s00105-016-3834-y

[6] Yu S Wan Y Wan Q Chinese expert consensus on diagnosis and treatment of postherpetic neuralgia. Chin J Pain Med 2016;22:161–7.

[7] Tang Wei Ll The discussion on postherpetic neuralgia treated by “Removing the Stagnation of Fire”. J Acupunct Moxibustion 2010;26:19–21.

[8] Lin Shiyu Li Jingjing Pei Wenya Summary of the origin and application of Lingnan fire needle. J Acupunct Moxibustion 2017;33:69–71.

[9] Jiao Danhong PJ Cao J 30 cases of herpes zoster treated by fire needle plus cupping. Chin J Dermatol Venereol 1994;2:119.

[10] Zhang Ying Liang Zuohui Liu Xiuhong Efficacy observation of fire needle Zan needling method in the treatment of 30 cases of acute herpes zoster. Yunnan J Tradit Chin Med 2016;39:50–3.

[11] GRADEpro GDT: GRADEpro Guideline Development Tool [Software]. McMaster University, 2015 (developed by Evidence Prime, Inc.). Available from gradepro.org.

[12] Kelin H Tingting L Ruijie M Treatment of 32 cases of acute herpes zoster with Jiaji fire needle and Ashi point acupuncture. Zhejiang J Tradit Chin Med 2019;54:842.

[13] Yongnan J Jianhui G Qinglin W Treatment of 42 cases of acute herpes zoster with fire needle and cupping. Tradit Chin Med Clin Res 2019;11:95–7.

[14] Xinghua G Chaoyang M Ying G Clinical observation on the treatment of herpes zoster with fire needle combined with electroacupuncture and its effect on serum IL-4 and TNF-α. Liaoning J Tradit Chin Med 2019;46:2399–404.

[15] Qianglin N Clinical study on the treatment of middle-aged and elderly herpes zoster with fibro-fire needle combined with surrounding acupuncture. Shenzhen J Integr Tradit Chin West Med 2020;30:57–8.

[16] Shamseer L Moher D Clarke M Preferred reporting items for systematic review and meta-analysis protocols (PRISMA-P) 2015: elaboration and explanation. BMJ 2015;350:g7647 DOI 10.1136/bmj.g7647. 25555855 10.1136/bmj.g7647

[17] Thyregod HG Rowbotham MC Peters M Natural history of pain following herpes zoster. Pain 2007;128:148–56. 17070998 10.1016/j.pain.2006.09.021 PMC1905461

[18] Higgins JPT Altman DG Gøtzsche PC The Cochrane Collaboration's tool for assessing risk of bias in randomised trials. BMJ 2011;343:d5928 DOI 10.1136/bmj.d5928. 22008217 10.1136/bmj.d5928 PMC3196245

[19] Review Manager (RevMan) [Computer program]. Version 5.3.5. Copenhagen: The Nordic Cochrane Centre, The Cochrane Collaboration. 2014.

[20] Trial Sequential Analysis (TSA) [Computer program]. Version 0.9.10 Beta. Copenhagen: Copenhagen Trial Unit. 2017.

[21] Rosamilia LL Herpes zoster presentation, management, and prevention: a modern case-based review. Am J Clin Dermatol 2020;21:97–107. DOI 10.1007/s40257-019-00483-1. 31741185 10.1007/s40257-019-00483-1

